# Variation Between Multidisciplinary Tumor Boards in Clinical Staging and Treatment Recommendations for Patients With Locally Advanced Non-small Cell Lung Cancer

**DOI:** 10.1016/j.chest.2020.07.054

**Published:** 2020-07-30

**Authors:** Fieke Hoeijmakers, David J. Heineman, Johannes M. Daniels, Naomi Beck, Rob A.E. M. Tollenaar, Michel W.J. M. Wouters, Perla J. Marang-van de Mheen, Wilhelmina H. Schreurs, MDT Study Group, Nicole P. Barlo, Bart P.C. Hoppe, Wouter Jacobs, Robin Cornelissen, Joost D.J. Janssen, Sietske A. Smulders, Niels J.M. Claessens, Susan C. van ‘t Westeinde, Steven R. Rutgers, Franz M.N. H. Schramel

**Affiliations:** a Department of Surgery, Leiden University Medical Center, Leiden, The Netherlands; b Department of Biomedical Data Sciences, Medical Decision Making, Leiden University Medical Center, Leiden, The Netherlands; c Scientific Bureau, Dutch Institute for Clinical Auditing, Leiden, The Netherlands; d Department of Surgery, Amsterdam UMC, VU University Medical Center, Amsterdam, The Netherlands; e Department of Cardiothoracic Surgery, Amsterdam UMC, VU University Medical Center, Amsterdam, The Netherlands; f Department of Pulmonary Diseases, Amsterdam UMC, VU University Medical Center, Amsterdam, The Netherlands; g Department of Surgical Oncology, The Netherlands Cancer Institute / Antoni van Leeuwenhoek, Amsterdam, The Netherlands; h Department of Surgery, Northwest Clinics, Alkmaar, The Netherlands

**Keywords:** multidisciplinary team, multidisciplinary tumor board, non-small cell lung cancer, staging, treatment, EBUS, endobronchial ultrasound, κ_free_, Randolph’s free-marginal multirater kappa, MDT, multidisciplinary tumor board, NSCLC, non-small cell lung cancer

## Abstract

**Background:**

Accurate diagnosis and staging are crucial to ensure uniform allocation to the optimal treatment methods for non-small cell lung cancer (NSCLC) patients, but may differ among multidisciplinary tumor boards (MDTs). Discordance between clinical and pathologic TNM stage is particularly important for patients with locally advanced NSCLC (stage IIIA) because it may influence their chance of allocation to curative-intent treatment. We therefore aimed to study agreement on staging and treatment to gain insight into MDT decision-making.

**Research Question:**

What is the level of agreement on clinical staging and treatment recommendations among MDTs in stage IIIA NSCLC patients?

**Study Design and Methods:**

Eleven MDTs each evaluated the same 10 pathologic stage IIIA NSCLC patients in their weekly meeting (n = 110). Patients were selected purposively for their challenging nature. All MDTs received exactly the same clinical information and images per patient. We tested agreement in cT stage, cN stage, cM stage (TNM 8th edition), and treatment proposal among MDTs using Randolph’s free-marginal multirater kappa.

**Results:**

Considerable variation among the MDTs was seen in T staging (κ, 0.55 [95% CI, 0.34-0.75]), N staging (κ, 0.59 [95% CI, 0.35-0.83]), overall TNM staging (κ, 0.53 [95% CI, 0.35-0.72]), and treatment recommendations (κ, 0.44 [95% CI, 0.32-0.56]). Most variation in T stage was seen in patients with suspicion of invasion of surrounding structures, which influenced such treatment recommendations as induction therapy and type. For N stage, distinction between N1 and N2 disease was an important source of discordance among MDTs. Variation occurred between 2 patients even regarding M stage. A wide range of additional diagnostics was proposed by the MDTs.

**Interpretation:**

This study demonstrated high variation in staging and treatment of patients with stage IIIA NSCLC among MDTs in different hospitals. Although some variation may be unavoidable in these challenging patients, we should strive for more uniformity.

Lung cancer is the leading cause of cancer-related death worldwide.^1^ New and better imaging methods, such as PET-CT and endobronchial ultrasound (EBUS) or endoscopic ultrasound, have been implemented over the last decades. Furthermore, a rapidly expanding array of treatment options has become available. Although lung cancer treatment has been a disappointing endeavor in terms of overall survival in the past decades,^2^^,^^3^ evidence shows that survival is improving.^4^ This development should prompt the medical community relentlessly to try to improve lung cancer care further.

**Take-home Points**

**Study Question**: What is the level of agreement on clinical staging and treatment recommendations among MDTs in challenging stage IIIA NSCLC patients?

**Results**: Intermediate agreement was found among the MDTs in TNM staging and treatment recommendations with κ values of 0.53 (0.35-0.72) and 0.44 (0.32-0.56), respectively.

**Interpretation**: A high variation was found among MDTs in staging and treatment recommendations for patients with stage IIIA NSCLC. Although some variation may be unavoidable in these challenging patients, we should strive for more uniformity.

Accurate clinical staging is necessary to determine the best treatment strategy for the individual patient, particularly with expanding treatment options like stereotactic ablative radiotherapy and different types of induction therapy (eg, immunotherapy). Staging often consists of a combination of imaging methods and (minimally) invasive staging procedures. Although these separate methods show high sensitivity and specificity,^5^ concordance between clinical and pathologic stage of NSCLC is surprisingly low, between 50% and 60%.^6^ Low staging concordance combined with expanding treatment options also may result in differences between hospitals, especially in locally advanced disease, when staging accuracy is relatively low and clear evidence for treatment options is lacking.^6^ Accurate diagnosis and staging are particularly important in stage IIIA patients, because it may influence their chance of allocation to curative-intent treatment.

The multidisciplinary team or multidisciplinary tumor board (MDT) has a crucial role in clinical staging and proposing the primary treatment. The literature shows that MDT recommendations change the initial treatment plan in 40% of lung cancer patients, and some studies even show clinically relevant overall survival benefits.^7^^,^^8^ Dutch guidelines require that decisions regarding the treatment of lung cancer patients be made by an MDT and strive for uniform treatment nationwide.^9^ In 2017, the percentage of NSCLC patients discussed in an MDT meeting before curative treatment in The Netherlands was 98.9%.^10^ The objective of this study was to determine the level of agreement among MDTs regarding clinical staging and treatment recommendations for patients with stage IIIA NSCLC to gain insight into MDT decision-making.

## Methods

### Multidisciplinary Tumor Boards

Lung oncology MDTs from 26 hospitals were invited to participate in this study among the total of 42 lung surgery-performing hospitals in The Netherlands. Per hospital, one oncology-specialized pulmonologist—preferably the MDT chair—was approached and asked to participate in the study. This pulmonologist informed the MDT and, as a representative for the entire MDT, provided written consent to participate. The selected hospitals were sampled to represent both academic and peripheral hospitals and both low- and high-volume centers, geographically well dispersed over The Netherlands. Using Dutch Lung Cancer Audit for Surgery data, hospital volume was determined by calculating the mean number of annual oncologic parenchymal lung resections from 2013 through 2015.^11^ We defined low-volume hospitals as those performing 20 to 49 anatomic parenchymal resections per year and high-volume hospitals as those performing 50 or more anatomic parenchymal resections per year (fewer than 20 lung resections per year is not allowed according to the Dutch quality standards [www.soncos.org]).

### Patients

Patients were accrued from 5 hospitals (of which 4 also participated in this study) representing Dutch lung cancer care practice in general regarding volume, teaching status, and geographic area. All of the 2014 and 2015 patients who underwent surgery for locally advanced NSCLC (defined as pathologic stage IIIA regarding the TNM classification, 7th edition^12^) were traced using the Dutch Lung Cancer Audit for Surgery. Stage IIIA patients were chosen because in this diverse patient group, staging can be challenging regarding the N status and invasion in surrounding structures and several treatment strategies are possible without clear evidence on which strategy results in the best outcomes. The primary investigator (F. H.) visited these hospitals to investigate patient files and to collect additional patient and diagnostic (imaging) information. Together with the local lung surgeon, it was decided whether patients were suitable for the study. Factors considered in this decision were the possibility of multidisciplinary discussion about tumor stage, nodal stage, and different treatment options. Also, sufficient information regarding history and imaging had to be available. All patients considered suitable were discussed by the primary investigator and two lung surgeons (D. J. H. and W. H. S.). Ultimately, 10 patients with a variety in staging and treatment difficulties were selected from five hospitals. All patient information and imaging were stripped of data directly traceable to the patient to ensure their privacy.

### Study Design

The pulmonologists of participating hospitals were asked to include the 10 patients, one or two each time, into their weekly MDT meeting within a period of 4 to 6 months. Case material included full patient history, diagnostic reports (conclusions in case of imaging, full reports in case of endoscopic procedures or bronchoscopy), and images (sent to the MDT on a DVD) available before the start of treatment and necessary to decide on clinical stage and treatment. Patients were presented as second-opinion referrals and in a way that they could be distinguished as little as possible from actual patients.

MDTs were asked to propose the clinical TNM stage (8th edition, implemented nationwide for lung cancer from January 1, 2017) and to propose a treatment plan. The pulmonologist introducing the study patients scored the proposed clinical stage and the treatment recommendation, and completed a short questionnaire about the choices considered during the MDT meeting for each patient (e-Appendix 1).

### Outcomes

Primary outcomes were the level of agreement among MDTs regarding (1) clinical TNM stage and (2) treatment recommendations. Secondary outcomes were variation in proposal of extra diagnostics and adherence to the Dutch staging guidelines.^9^

### Statistical Analysis

Randolph’s free-marginal multirater kappa (κ_free_) was used to measure agreement.^13^ It is a chance-adjusted measure of agreement for any number of patients, categories, or raters that can be used when raters are not forced to assign a certain number of patients to each category. A κ value of less than 0.40 is considered poor, one between 0.40 and 0.75 is considered intermediate to good, and one of more than 0.75 is considered excellent. To avoid overestimation of agreement, expressed as a high κ value, we reduced the categories for each outcome.^14^ Therefore, subgroups of T stage, M stage, overall TNM stage, and treatment were combined (eg, T1a, T1b, and T1c became T1), and in case of doubts by the MDT expressed as the proposal of two stages, the highest stage was chosen (eg, stage T3/4 became stage T4). This was chosen because we hypothesized that treatment and diagnostic plans are made based on the highest stage. For example, when doubt exists regarding whether the T stage is T2 or T3, induction therapy probably would be proposed, given the suspicion of the T3 stage. Furthermore, when doubt existed between stage N2 or N1, the extra diagnostics usually would be advised based on the suspicion of N2 disease. If participating hospitals that initially treated selected patients could not review these patients because of technical or other issues, the originally proposed clinical stage and received treatment were used for the analyses.

### Approval

**Figure 1:**
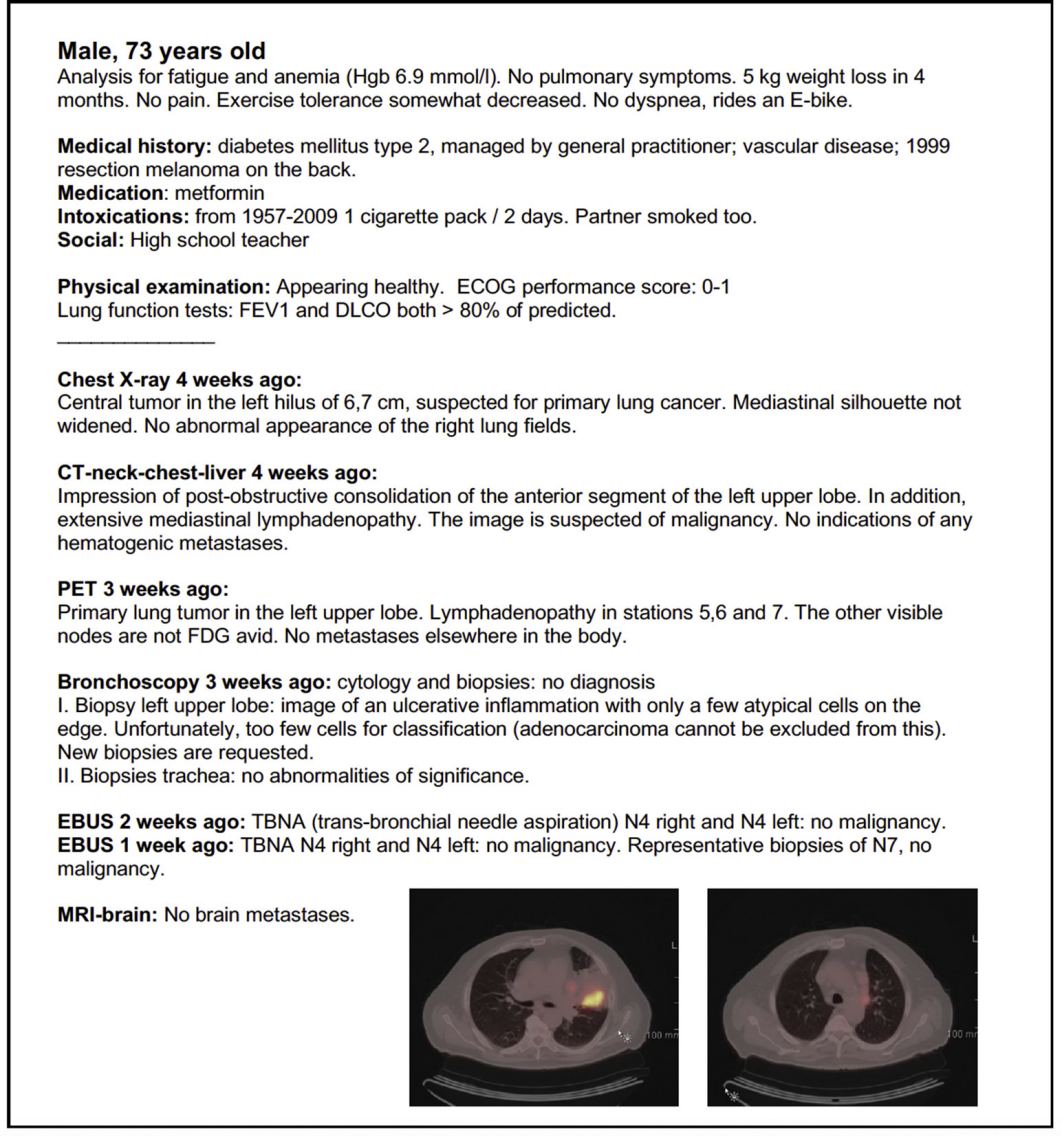
Example of the clinical information and imaging from one of the patients provided to all MDTs. ECOG = Eastern Cooperative Oncology Group; FDG = fluorodeoxyglucose; MDT = multidisciplinary tumor board.

The Medical Ethics Committee of the Leiden University Medical Center approved this study (project number, G16.097). No informed consent from patients was needed because of anonymized patient and image acquisition. For one of the published patients in this article (Fig 1), written informed consent was obtained from the patient.

## Results

### Multidisciplinary Tumor Boards

Pulmonologists from 26 hospitals were approached to participate. Sixteen MDTs agreed to participate, and ultimately 11 MDTs completed all patient reviews and were included for analysis. Three MDTs were from academic hospitals and eight were from peripheral hospitals. Five high-volume and six low-volume hospitals participated in the study. Overall, these hospitals and their MDTs were a good representation of Dutch practice. Some MDTs were linked by teleconferencing for consultation with a representative of an also-participating (academic) reference center. In such cases, the consulted representatives were asked to give input only in their own hospital. All MDTs reviewed the patients between September 2017 and December 2018.

### Patients

Characteristics of all patients are shown in Table 1. An example of one of the presented patients (patient 4) is shown in Figure 1. Patients 4 and 7—collected from two participating hospitals—were not reevaluated by these hospitals because of technical errors concerning uploading of images. The original clinical stage and treatment proposal from the initial presentation to the respective MDT were used for these patients.

**Table 1:** Patient and Tumor Characteristics of Patients Presented to the MDT

	Patient 1	Patient 2	Patient 3	Patient 4	Patient 5	Patient 6	Patient 7	Patient 8	Patient 9	Patient 10
Presented to the MDT										
Age (y)	65	49	60	73	66	77	75	66	64	77
Sex	Male	Female	Female	Male	Male	Male	Male	Male	Female	Male
Initial symptoms	Blood in phlegm	Coughing up blood	Thoracic pain	Fatigue and anemia	Cough and blood in phlegm	Cough and phlegm	Blood in phlegm	Cough and phlegm	Fatigue	Anomaly at follow-up EVAR
Tumor location	Central tumor left lower and middle lobe 9.4 cm	Mass left suprahilar, compressing the upper main bronchus	Tumor left lower lobe 6.3 cm + satellite lesion	Tumor left upper lobe with postobstructive consolidation	Tumor left upper lobe, 10 cm	Suspected tumor left upper lobe, possible mediastinal invasion	Large tumor left lower lobe	Tumor left upper lobe, possible mediastinal and pericardial invasion	Tumor 4.5 cm left upper lobe	2.5 cm tumor left upper lobe
Histologic type	No preoperative biopsy	Squamous cell carcinoma	Non-small cell lung cancer	No representative biopsy	Non-small cell lung cancer	Squamous cell carcinoma	Adenocarcinoma	Non-small cell lung cancer	Non-small cell lung cancer	Unknown
Suspected N metastases	Hilar nodes can not be distinguished from primary tumor, no suspected mediastinal nodes	Pathologically proven micrometastasis nodal station 4R	PET: suspected hilar node left; EBUS N10: metastasis NSCLC	FDG-avid nodal stations 5, 6, and 7. EBUS with punction of N4R, N4L, and 7: no malignancy	Very avid left hilar nodes; small FDG activity in 2R, 4R, 3A, and 7. EBUS 2R, 4R, 7: not suspected, no malignancy; 10R suspected, punction failed	FDG-avid mediastinal nodes, dd inflammation, metastases; EBUS 4R: not suspected, lymphoid tissue	Suspected hilar node and FDG uptake in node supraclavicular; EBUS hilar node: positive results; cytologic examination supraclavicular: inflammation	N10 left: nodal metastases	Necrotic hilar lymph nodes and PET suspected nodes station 5/6; EUS 4L suspected; punction 4L and 5: mainly blood, few lymphoid cells	Hilar en paratracheal nodes suspected on CT/PET; EBUS 4R and mediastinoscopy 4L, 4R, and 7: no malignancy
Suspected M metastases	Pleural lesion left dd pleural carcinomatosis	No	Small nodule left lung, not FDG avid	No	No	Small node left adrenal gland, not FDG avid	No	No	No	No
PET/CT	Both	Both	Both	Both	Both	Both	Both	Both	Both	Both
EUS/EBUS	No	No	EBUS	EBUS	EBUS	EBUS	EBUS hilar node	No	EUS	EBUS
Other diagnostics	Pleural punction	Bronchoscopy, mediastinoscopy, brain MRI	None	Bronchoscopy, brain MRI	Transthoracic punction	Transthoracic punction	Punction supraclavicular node	Transthoracic punction; ultrasound heart: no invasion	CT brain, bronchoscopy	Brain MRI, mediastinoscopy
Postoperative staging										
pT	Tumor 9.0 cm, invasion in visceral pleura and pericardium	Tumor 2.4 cm	Tumor 7.9 cm	Tumor 10.0 cm	Tumor 9 cm, chest wall invasion	Tumor 5.1 cm, chest wall invasion	Tumor 9.8 cm	Tumor 4.9 cm, invasion in pericardium	2 separate tumor nodules, diameters 3 and 5 cm, pleural invasion at the hilus	Tumor 2.7 cm
PN	Hilar lymph node metastases	Lymph nodes 4R positive	Hilar lymph node metastases	Lymph nodes 4L, 5, and 6: 3/6 positive	10R + 11R positive, 4R positive	Positive lymph nodes in chest wall	Direct invasion in hilar lymph node	Positive hilar lymph nodes and 4R + 7	Positive lymph nodes stations 6 and 12	Positive lymph nodes 4R
pTNM7	pT3N1M0	pT1bN2M0	pT3N1M0	pT3N2M0	pT3N2M0	pT3N1M0 (T3 based on chest wall invasion)	pT3N1M0	pT3N2M0 (based on pericardial invasion)	pT3N2M0 (T3 based on 2 separate tumor nodules)	pT1bN2M0
pTNM8	pT4N1M0 (based on tumor > 7 cm)	pT1cN2M0 (based on a tumor of 2.4 cm)	pT4N1M0 (based on tumor > 7 cm)	pT4N2M0 (based on tumor > 7 cm)	pT4N2M0 (based on tumor > 7 cm)	pT3N1M0 (T3 based on chest wall invasion and tumor > 5 cm)	pT4N1M0 (based on tumor > 7 cm)	pT3N2M0	pT3N2M0	pT1cN2M0
Histologic type	Adenocarcinoma	Squamous cell carcinoma	Adenocarcinoma	Adenocarcinoma	Adenocarcinoma	Squamous cell carcinoma	Adenocarcinoma	Adenosquamous carcinoma	Adenocarcinoma	Adenocarcinoma

dd = differential diagnosis; EBUS = endobronchial ultrasound; EUS = endoscopic ultrasound; EVAR = endovasculair aneurysm repair; FDG = fluorodeoxyglucose; L = left; MDT = multidisciplinary tumor board; R = left.

### Primary Outcomes

#### Staging

Variation among MDTs in both T and N staging was considerable (Table 2). No consensus was found regarding clinical T stage in 8 of 10 patients. Most variation in T stage was seen in patients with possible invasion of surrounding structures, such as the pericardium or mediastinum. In the questionnaire, MDTs reported that this was the most frequently encountered point of discussion (10% of observations), followed by tumor size (2.8%). Variation in clinical N stage occurred in 7 of 10 patients. For most patients, variation occurred between N0 and N1 or between N1 and N2, but in some patients, staging proposals ranged from N0 to N2. Distinction between N1 and N2 disease was mentioned most frequently by MDTs as a point of discussion (17% of observations). Although considerable variation was present between N0 and N1 in some of the patients, this was mentioned rarely as a discussion point (< 1%). Regarding the M stage, variation occurred in 2 of 10 patients. Percentages of overall agreement for T, N, M and overall TNM stage were 67.8%, 67.3%, 91.8%, and 63.5%, respectively. The levels of agreement using κ_free_ were 0.57 (CI, 0.40-0.74), 0.56 (CI, 0.35-0.78), 0.88 (CI, 0.71-1.00), and 0.54 (CI, 0.36.-0.73), respectively, indicating intermediate to good agreement except for the excellent agreement regarding M stage.

**Table 2:** Proposed Clinical TNM Stage, Additional Diagnostics, and Treatment Recommendations by All MDTs Per Patient

	Patient 1	Patient 2	Patient 3	Patient 4	Patient 5	Patient 6	Patient 7	Patient 8	Patient 9	Patient 10	Overall Agreement (%) κ_free_ Value
Original Clinical Stage^a^	cT4N1M0	cT2aN2M0	cT3N1M0	cT3N2M0	cT4N1M0	cT3/4N0/2M0	cT4N1M0	cT4N1M0	cT2bN0M0	cT1cN2M0	
Pathological stage^a^	pT4N1M0	pT1cN2M0	pT4N1M0	pT4N2M0	pT4N2M0	pT3N1M0	pT4N1M0	pT3N2M0	pT3N2M0	pT1cN2M0	
Clinical stage^a^											
T1	. . .	1	. . .	. . .	. . .	. . .	. . .	. . .	. . .	10	67.8 0.57 (0.40-0.74)
T2	. . .	7	. . .	3	. . .	1	. . .	. . .	9	1	
T3	. . .	. . .	9	8	. . .	6	2	8	2	. . .	
T4	11	3	2		11	4	9	3	. . .	. . .	
Nx	. . .	. . .	. . .	1	2	. . .	. . .	. . .	1	1	67.3 0.56 (0.35-0.78)
N0	7	. . .	. . .	. . .	. . .	5	. . .	. . .	. . .	3	
N1	4	1	11	. . .	7	. . .	10	11	1	5	
N2	. . .	10	. . .	10	2	6	1		9	2	
Mx	1	. . .	. . .		. . .	. . .	1	. . .	. . .	. . .	91.8 0.88 (0.71-1.00)
M0	8	11	11	11	11	11	9	11	11	11	
M1	2	. . .	. . .	. . .	. . .	. . .	1	. . .	. . .	. . .	
I	. . .	. . .	. . .	. . .	. . .	. . .	. . .	. . .	. . .	4	63.5 0.54 (0.36-0.73)
II	. . .	. . .	. . .	. . .	. . .	4	. . .	. . .	. . .	5	
IIIA	9^b^	9^b^	11^b^	3	7	2^b^	9^b^	11	9	2^b^	
IIIB	. . .	2	. . .	8^b^	4^b^	5	1	. . .^b^	2^b^	. . .	
IV	2	. . .	. . .	. . .	. . .	. . .	1	. . .	. . .	. . .	
Diagnostics											
EBUS (EUS)	6	. . .	. . .	. . .	1	. . .	4	5	. . .	2	. . .
Mediastinoscopy	1 (+2)	. . .	5	9	7 (+1)	6	4 (+2)	4 (+3)	9	. . .	. . .
Bronchoscopy	5	. . .	1	. . .	2	. . .	1	. . .	. . .	. . .	. . .
Transthoracic biopsy	. . .	. . .		5	. . .	. . .	. . .	. . .	1	. . .	. . .
Brain MRI	8	. . .	4	. . .	5	4	5	5	4	. . .	. . .
Other	7	. . .	. . .	. . .	2	2	3	2	4	5	. . .
No additional diagnostics	1	11	3	1	2	1	2	2	1	4	. . .
Treatment											
Induction therapy	1	7	. . .	. . .	6	7	1	2	2	. . .	57.3 0.43 (0.31-0.55)
Resection (after negative mediastinoscopy or EBUS results)	9	. . .	9	9	5	3	10	8	6	9	
Chemoradiotherapy	1	4	1	. . .	. . .	. . .	. . .	. . .	. . .	. . .	
Other	. . .	. . .	1	2	. . .	1	. . .	1	3	2	

Eleven MDTs each evaluated the same 10 patients. κ_free_ = Randolph’s free-marginal multirater kappa. See Table 1 legend for expansion of other abbreviations.

a Stages are adjusted from the 7th to the 8th edition of the TNM classification. Some tumors changed from stage IIIA to stage IIIB by adjustment to the TNM 8th edition classification.

b Pathologic stage (lung cancer was resected in all patients).

#### Treatment Recommendations

**Figure 2:**
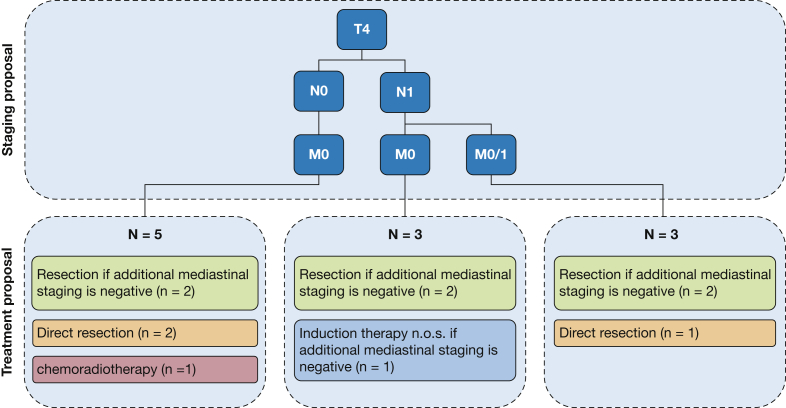
Staging and treatment tree for patient 1.

Regarding the variation in treatment proposal, a wide variety of recommendations was seen, with an overall agreement of 57.3% and κ_free_ of 0.43 (CI, 0.31-0.55) (Table 2). Definitive chemoradiotherapy was suggested only six times in total by all MDTs among 3 cases. All other recommendations included surgery or reconsidering surgery after induction therapy. Also notable was the variation seen in the use and type of induction therapy. Even among patients who received induction therapy, MDT recommendations varied between chemotherapy or chemoradiation (Table 3). Different staging results evidently can lead to different treatment recommendations. However, also within the same staging group, variation in treatment proposals was seen among all patients (Fig 2) (data of other patients not shown).

**Table 3:** Variation in Induction Therapy Recommendations

Pathologic Stage and Treatment	Patient 1	Patient 2	Patient 3^a^	Patient 4^a^	Patient 5	Patient 6	Patient 7	Patient 8	Patient 9	Patient 10^a^
pTNM8	pT4N1M0 (tumor > 7 cm)	pT1cN2M0	pT4N1M0 (tumor > 7 cm)	pT4N2M0 (tumor > 7 cm)	pT4N2M0 (tumor > 7 cm)	pT3N1M0 (chest wall invasion + tumor > 5 cm)	pT4N1M0 (tumor > 7 cm)	pT3N2M0	pT3N2M0	pT1cN2M0
Treatment	. . .	. . .	. . .	. . .	. . .	. . .	. . .	. . .	. . .	. . .
Induction n.o.s.	1	. . .	. . .	. . .	. . .	. . .	. . .	. . .	. . .	. . .
Induction chemotherapy	. . .	1	. . .	. . .	3	3	1	2	1	. . .
Induction radiotherapy	. . .	. . .	. . .	. . .	. . .	1	. . .	. . .	. . .	. . .
Induction CRT	. . .	6	. . .	. . .	2	3	. . .	. . .	1	. . .

CRT = chemoradiotherapy; n.o.s. = not otherwise specified. See Table 1 legend for expansion of other abbreviation.

a In Patients 3, 4, and 10, none of the MDTs recommended induction therapy.

### Secondary Outcomes: Additional Diagnostics and Guideline Adherence

A wide range of additional diagnostics was proposed by the MDTs (Table 2). In 8 of 10 patients (additional) invasive mediastinal staging—indicated in at least 7 of these 8 patients when strictly following the Dutch guidelines—was requested by more than half of the MDTs. MRI of the brain is considered necessary in locally advanced disease (all study patients) when considering curative-intent treatment, but was not performed in 7 of the 10 presented patients.^15^ Fewer than half of the MDTs requested MRI of the brain. In 25 of the 108 reassessments, no additional diagnostics were requested. For each patient, the proposed next step by the guideline was determined (F. H., D. J. H., and H. S.). This could be additional staging or treatment. In 79 of the 110 reviews, this step was proposed by the MDTs (71.8%). An MDT (possibly) withheld three different patients from optimal treatment. For two patients, this was done by not incorporating surgery into the treatment proposal, whereas in our opinion, both patients were suitable for surgery with or without neoadjuvant therapy. In the third patient, the MDT proposal was to start antibiotic and prednisolone treatment with reevaluation after 1 month.

## Discussion

The current study showed that clinical staging and treatment recommendations of patients with locally advanced NSCLC vary considerably among lung cancer MDTs in different hospitals in The Netherlands. The levels of agreement for both clinical staging and treatment recommendations mostly were less than 0.6, indicating only intermediate to good agreement among the different MDTs, except for M staging, for which an excellent level of agreement was found (κ_free_, 0.88). Treatment recommendations varied independently of staging. Agreement on treatment recommendations was lower than agreement on clinical staging.

### Variation in Staging and Additional Diagnostics

Variation in clinical T stage can be explained by different interpretations of imaging, which in turn generate different opinions regarding suspected invasion of surrounding structures. Another explanation could be measurement errors in tumor size. These have more impact when using the more detailed subdivision of the T classification in the 8th edition. Yet another explanation is that difficulties in interpretation of the TNM classification, for example, regarding the appropriate measurement of tumor size or separate tumor nodules, can account for variation in clinical T stage.^16^, ^17^, ^18^ Regarding clinical N staging, experience in interpreting imaging may be of influence, especially regarding the challenging task of interpreting fluorodeoxyglucose PET scans.^19^^,^^20^ Another factor of influence could be how specialists rely on endoscopic ultrasound or EBUS or on mediastinoscopy results, reflected in the amount of advised additional mediastinoscopies.^21^^,^^22^ In our selection of patients with locally advanced disease, conclusions about M stage were quite aligned.

Regarding the use of additional diagnostics, we found that (additional) invasive mediastinal staging, especially mediastinoscopy, was omitted regularly by MDTs. By indicating mediastinoscopy “if EBUS and/or EUS [endoscopic ultrasound] does not reveal nodal involvement in a situation of high clinical suspicion,”^15^ guidelines offer room for discussion on performing additional mediastinoscopy. However, one could discuss whether the amount of variation found among MDTs is desirable. In this regard, it is also important to be aware of the different indications for mediastinal staging; the risk of unforeseen N2 nodes is higher in clinical N1 or N2 nodes on PET-CT imaging compared with the risk of unforeseen N2 in fluorodeoxyglucose-negative tumors, central tumors, or tumors larger than 3 cm. Furthermore, EBUS has a low sensitivity compared with mediastinoscopy in clinical N1 disease.^23^ Some groups even advocate omitting EBUS in this patient group and perform only mediastinoscopy.^24^ Elsewhere, discussions are ongoing regarding whether to omit mediastinoscopy.^25^ Also, brain MRI was not requested when indicated by more than half of the MDTs, although it is advised by the Dutch guideline for stage III NSCLC patients considered for curative therapy. It is possible that some MDTs would have requested an MRI eventually, but nevertheless, it should have been discussed in the MDT meeting.^15^

### Variation in Treatment Recommendations

Treatment recommendations were studied independently of clinical staging. Reasons not to relate both to each other were (1) that all MDTs received exactly the same patient information and images; (2) that the treatment offered to the patient is the outcome most clinically relevant, regardless of the proposed clinical stage; and (3) that the TNM classification originally is prognostic, and although used for this purpose, it is not specifically meant for treatment decision-making, which is dependent on many other characteristics of the patient and the tumor.^26^ For example, the choice of whether to perform surgery also can depend on low pulmonary or cardiac function, patient age, or a large, necrotizing tumor that is not suitable for radiotherapy.

Regarding treatment recommendations, comparable variation was present among MDTs. Variation in treatment mainly was independent of clinical stage; other factors probably often played a larger role. Most variation was found concerning whether induction therapy was advised and the type of induction therapy. A possible explanation could be the lack of convincing evidence from randomized controlled trials regarding trimodality therapy and the heterogeneity of stage IIIA disease.^27^^,^^28^ Guidelines designate both definitive chemoradiotherapy and multimodality therapy including surgery as options for radical treatment and point out the importance of patient-tailored decision-making in experienced MDTs.^15^^,^^29^ The MDT should consider other factors for this group of heterogeneous tumors beyond (potential) resectability of the tumor. Also, patient factors like a history of interstitial lung disease and tumor characteristics such as cavitation and size play a role, especially in the tradeoff between surgery and radiotherapy. Different interpretations of these factors by the MDTs may have resulted in different therapeutic decisions. The variation found in this study underlines this recommendation of multidisciplinary discussion of these patients, but also shows that this does not avoid differences in treatment strategies among hospitals. Possible explanations for the variation could be differences in experience with treatments among hospitals or differences in attendance of medical specialists at MDT meetings, which has been shown previously by Lau et al,^30^ who found variation in resection rates to be related (partly) to the local availability of specialist thoracic surgeons. The variation in treatment that we found also was in line with a study by Tanner et al,^31^ who found a significant variation in treatment recommendations by oncologists for stage IIIA N2 NSCLC patients using hypothetical scenarios. Although incomparable in design, our multidisciplinary study using real-world patients and imaging, rather than hypothetical scenarios, showed that assessment by an MDT by itself does not result in equal treatment of patients, as we strive for in The Netherlands.

Besides practice variation, some of the suggested treatment proposals, in our opinion, withheld optimal treatment from the patient. Because of the heterogeneity of treatment options in locally advanced NSCLC and to prevent unwarranted practice variation, we suggest that difficult cases are discussed in or with an expert center. We expect that variation in treatment will increase in the near future because of the results of the PACIFIC trial, which showed prolonged survival with adjuvant durvalumab after chemoradiotherapy for unresectable stage III NSCLC.^32^ Especially when doubts exist about resectability and invasion of vital structures, some MDTs probably will choose this strategy in the near future. As the role of immunotherapy in stage III NSCLC continues to evolve, it probably will be incorporated in the treatment of all stage III patients in the future in the adjuvant or neoadjuvant setting, or both (ie, also together with surgery). As a result, stage III patients will remain an interesting challenge for MDTs.

### MDT Expertise

The variation found in this study underlines the importance of evaluation of NSCLC patients with locally advanced disease in an MDT setting. For the individual patient, this is of additional value because of clustering of expertise among different medical specialists and individualization of treatment plans. We nevertheless have to remain critical about these MDT meetings as the ultimate instrument for quality assurance in individual patients. More uniformity could be gained with more up-to-date guidelines and sharing knowledge of recent developments in the literature among MDTs, for example, in consensus meetings organized at a regional or national level. It also seems that the available expertise, beliefs, and attitude can vary considerably, depending on the personal experiences of individual specialists or even an entire department. The latter especially could create a “force field” within an MDT, resulting in more conservative or invasive treatment. Assuring that the available expertise, which can vary based on the joint expertise of the medical specialists participating in an MDT, is on a similar level could reduce variation in the quality of staging and treatment decisions.

In the light of uncertainty about the most effective treatment strategies, variation is not necessarily a bad thing. Depending on the specific expertise of hospitals, but also on the preferences of patients, sometimes one treatment is considered more appropriate in a specific case and sometimes the other (eg, [chemo]radiotherapy or resection). However, in our opinion, this study underlined that in cases where MDT advice is hardly justified by scientific evidence, a second opinion from another MDT could be considered. Besides, we must continue to investigate better diagnostic methods and strategies or incorporate more expertise in assessing the results of these diagnostics, for example, with the support of registry data and analytic techniques like machine learning or artificial intelligence.

Research is needed to gain more insight into the decision-making process and, we hope, will create opportunities to improve patient-tailored decisions. To be able to do so, real-world data from registries are essential to evaluate treatment patterns, but are probably not enough. To gain a good understanding of the choices made, detailed information is needed, for example, by keeping track of patients not staged or treated by guidelines and by registering reasons (when and why) for guideline deviation. In the event of unjustified guideline deviation, an improvement process can be deployed using a plan-do-check-act cycle to improve quality of care. To do so optimally, patients with locally advanced NSCLC are ideally discussed in, or at least with, a team or center with comprehensive expertise.

### Strengths and Limitations

The major strength of this study is the design in which lung cancer patients were evaluated in the clinical setting of the MDT, one or two per session, to guarantee the normal MDT routine as much as possible. Providing the MTDs with exactly the same information of real patients, including imaging, made a reliable comparison possible.

Concerning limitations, in most of the patients, additional diagnostics were requested by MDTs, which probably resulted in a preliminary treatment plan. This could have increased the variation in treatment recommendations that was found, whereas variation of final treatments given may have been smaller. Another factor that could have resulted in increased variation in our study compared with routine practice is missing data. Although patients were chosen to be complete regarding their history, diagnostic reports, and imaging, and all hospitals were provided with the same information, that in some cases assumptions were made by MDTs about factors or information considered important, but not available, could not be avoided. This might have led to different assumptions by MDTs regarding staging conclusions and treatment recommendations. Nevertheless, for all patients, all information to follow treatment guidelines was available. However, regarding discussion study patients, MDTs also could have followed guidelines more closely than they usually do.

Patients were selected based on the possible discussion about staging, and therefore were not fully representative for all stage IIIA NSCLC patients. Because of this purposive selection, variation in staging could be expected beforehand. The variation found in this study therefore is likely representative for a selected group of patients with locally advanced disease, and not directly generalizable to all patients discussed in an MDT.

## Conclusions

Our study showed substantial variation in clinical staging conclusions and treatment recommendations by MDTs for stage IIIA NSCLC patients. Available guidelines do not prevent this variation. Further research should gain a better understanding of what happens in an MDT as well as which variation is undesirable and how to influence this. Robust evidence on strategies resulting in better outcomes is needed, and regional collaboration could lead to more uniform treatment of patients.

## Supplementary Data

e-Online Data
