# Supplementary material for: Variation Between Multidisciplinary Tumor Boards in Clinical Staging and Treatment Recommendations for Patients With Locally Advanced Non-small Cell Lung Cancer
Source: Chest. 2020 Jul 30;158(6):2675–87. doi: 10.1016/j.chest.2020.07.054 (PMC7768935; doi:10.1016/j.chest.2020.07.054)
Supplement: e-Online Data [file mmc1.pdf]

CHEST Online Supplement

Variation Between Multidisciplinary Tumor Boards in Clinical Staging and Treatment Recommendations for Patients With Locally Advanced Non-small Cell Lung Cancer

Fieke Hoeijmakers, MD; David J. Heineman, MD, PhD; Johannes M. Daniels, MD, PhD; Naomi Beck, MD; Rob A.E. M. Tollenaar, MD, PhD; Michel W.J. M. Wouters, MD, PhD; Perla J. Marang-van de Mheen, PhD; and Wilhelmina H. Schreurs, MD, PhD; on behalf of the MDT Study Group

CHEST 2020; 158(6):2675-2687

Online supplements are not copyedited prior to posting and the author(s) take full responsibility for the accuracy of all data.

© 2020 AMERICAN COLLEGE OF CHEST PHYSICIANS. Reproduction of this article is prohibited without written permission from the American College of Chest Physicians. See online for more details. **DOI:** 10.1016/j.chest.2020.07.054

e-Appendix 1.

MDT-study – Questionnaire cases **Name patient: ______________________**

Please circle what applies.

Which specialists are present (number):

- Pulmonologist	1x	2x	3x	4x	5x
- Surgeon	1x	2x	3x	4x	5x
- Radiotherapist	1x	2x	3x	4x	5x
- Radiologist	1x	2x			
- Nuclear medicine physician	1x	2x			
- Pathologist	1x	2x			
- Clinical nurse specialist	1x	2x	3x	4x	5x
- (and/or case manager/oncology nurse)					
- Other: ____					

Is there discussion about:

- T-stage? yes / no In case yes, about what?

____

- N-stage? yes / no In case yes, about what?

____

- M-stage? yes / no In case yes, about what?

____

- Treatment plan? yes / no In case yes, about what?

____

Are further diagnostics requested? yes / no

In case yes, which diagnostics: ____

Conclusion

**Stage (according TNM8):** TX	T0	T1(mi)	T1a	T1b	T1c	T2a	T2b	T3	T4
NX	N0	N1	N2	N3					
M0	M1a	M1b	M1c						

Proposed treatment plan:____

____

(In case additional diagnostics are requested, please also state whether / which preliminary treatment plan has been proposed)

Any other comments /considerations: ________________________________

____

201387

Online supplements are not copyedited prior to posting and the author(s) take full responsibility for the accuracy of all data.
